# Anomalies in Dopamine Transporter Expression and Primary Cilium Distribution in the Dorsal Striatum of a Mouse Model of Niemann-Pick C1 Disease

**DOI:** 10.3389/fncel.2019.00226

**Published:** 2019-05-24

**Authors:** Micaela Lucarelli, Chiara Di Pietro, Gina La Sala, Maria Teresa Fiorenza, Daniela Marazziti, Sonia Canterini

**Affiliations:** ^1^Division of Neuroscience, Department of Psychology, Center for Research in Neurobiology ‘Daniel Bovet’, Sapienza University of Rome, Rome, Italy; ^2^PhD Program in Behavioral Neuroscience, Sapienza University of Rome, Rome, Italy; ^3^Institute of Cell Biology and Neurobiology, Italian National Research Council, Rome, Italy

**Keywords:** Niemann-Pick C1, mouse model, striatum, primary cilium, dopamine

## Abstract

The Niemann-Pick type C1 (NPC1) is a rare genetic disease characterized by the accumulation of endocytosed cholesterol and other lipids in the endosome/lysosome compartments. In the brain, the accumulation/mislocalization of unesterified cholesterol, gangliosides and sphingolipids is responsible for the appearance of neuropathological hallmarks, and progressive neurological decline in patients. The imbalance of unesterified cholesterol and other lipids, including GM2 and GM3 gangliosides, alters a number of signaling mechanisms impacting on the overall homeostasis of neurons. In particular, lipid depletion experiments have shown that lipid rafts regulate the cell surface expression of dopamine transporter (DAT) and modulate its activity. Dysregulated dopamine transporter’s function results in imbalanced dopamine levels at synapses and severely affects dopamine-induced locomotor responses and dopamine receptor-mediated synaptic signaling. Recent studies begin to correlate dopaminergic stimulation with the length and function of the primary cilium, a non-motile organelle that coordinates numerous signaling pathways. In particular, the absence of dopaminergic D2 receptor stimulation induces the elongation of dorso-striatal neuron’s primary cilia. This study has used a mouse model of the NPC1 disease to correlate cholesterol dyshomeostasis with dorso-striatal anomalies in terms of DAT expression and primary cilium (PC) length and morphology. We found that juvenile *Npc1^nmf*164*^* mice display a reduction of dorso-striatal DAT expression, with associated alterations of PC number, length-frequency distribution, and tortuosity.

## Introduction

Niemann-Pick type C1 (NPC1) is a rare lysosomal lipid storage disorder caused by mutations in the *NPC1* gene, whose protein mediates the egress of cholesterol from lysosomes/endosomes (Peake and Vance, 2010). NPC1 patients develop severe neurological-neurovisceral disorders, including cerebellar ataxia, dysarthria, dysphagia, seizures, and progressive dementia (Vanier, 2010, 2013).

Niemann-Pick type C1 cells display defective synthesis and mobilization of endocytosed cholesterol to the plasma membrane, which affects the functions of neurotransmitters, and their receptors (Fiorenza et al., 2013, 2018).

Dopamine transporter (DAT) regulates the spatio/temporal dynamics of dopamine (DA) neurotransmission by regulating the reuptake of extracellular DA into presynaptic terminals. DAT localization and function are directly regulated by cholesterol (Jones et al., 2012). For instance, cholesterol depletion studies have shown that lipid rafts regulate DAT cell-surface expression (Foster et al., 2008; Gabriel et al., 2013), and modulate its activity (Adkins et al., 2007). In addition, cholesterol has been shown to stabilize DAT conformation and DA binding (Hong and Amara, 2010). A number of G protein-coupled receptors including dopaminergic D2-receptors (D2R) are localized to the primary cilium (PC) of mammalian neurons and the lack of D2 dopaminergic input increases striatal PC length (Marley and von Zastrow, 2010; Miyoshi et al., 2014).

We have recently reported an impairment of Sonic hedgehog (Shh) signaling and PC density and length, in hippocampal neurons of *Npc1*^−/−^ mice and fibroblasts of NPC1 patients (Canterini et al., 2017). PC is a non-motile organelle that plays critical roles in coordinating numerous neuronal/developmental signaling pathways. Alterations of PC morphology and localization are responsible for ciliopathies, disorders that manifest, like the NPC1 disease, a constellation of clinical features including ataxia, retinal degeneration, behavioral disturbance, and intellectual disability (Waters and Beales, 2011; Guo et al., 2015).

In this study we investigate DA signaling and reception in the striatum of mouse of the *Npc1^nmf*164*^* strain, that bears a point mutation in the *Npc1* gene (D1005G), resulting in a milder, and late-onset form comparable to the most part of human cases (Maue et al., 2012). In this study, we show that juvenile *Npc1^nmf*164*^* mice display a reduction of DAT expression and alteration of PC number and length-frequency distribution in the dorsal striatum.

Our findings identify early and subtle anomalies in striatal dopaminergic neurotransmission that might contribute to the subsequent appearance of NPC1 disease manifestations.

## Materials and Methods

### Animals and Treatments

Homozygous *Npc1^nmf*164*^* mice maintained on BALB/cJ background were derived from heterozygous matings. Genotypes were identified by PCR analysis of tail DNA (Palladino et al., 2015).

Animal experimental protocols and related procedures were approved by the Italian Ministry of Health-General Directorate of Animal Health (995/2016; D.Igs. 26/2014). All efforts were made to minimize animal suffering, according to European Directive 2010/63/EU.

### Tissue Dissection

Brains of postnatal (PN) day 30 *Npc1^nmf*164*^* mice and wild-type (*wt*) littermates (5 mice/genotype) (Supplementary Table S1) were collected on ice-cold PBS and cut along the mid-sagittal plane. For Western Blotting, punches of the dorsal striatum (DS) were obtained from coronal slices of one hemisphere using a steel needle (1.5 mm diameter) (Colelli et al., 2010; Campus et al., 2017). The other hemisphere was fixed overnight in 4% paraformaldehyde and cut on Leica-Vibratome (S1000, Leica), for immunohistochemistry (IHC).

### Western Blot Analysis

Tissue punches of DS were processed for protein extraction and Western blot analysis as previously described (Di Pietro et al., 2017). Primary and secondary antibodies used are listed in Supplementary Table S2. Band intensity was normalized to α-tubulin signal. The average values were expressed in arbitrary units, as a ratio to *wt* mean values.

### IHC of Primary Cilium Markers and Measurements

Double IHC staining on free-floating sections (30 μm) was performed as previously described (Canterini et al., 2012). Primary and secondary antibodies data are listed in Supplementary Table S2.

Only striatal PC “clearly” double-stained for γ-tubulin (basal-body) and ACIII (PC-shaft) were selected for morphological analysis using Neurolucida analysis system (MBF Bioscience, Williston, VT, United States), connected to Olympus BX53 microscope (100X/1.25 numerical aperture) with 40X/100X immersion objective lens. An average of 6 PC was measured from 10 random fields per mice (*n* = 318 PC/genotype).

### Statistics

A Mann-Whitney *U*-test was used to determine the difference in protein levels (GraphPad Software, Inc). The D’Agostino & Pearson omnibus normality test was used to assess the distribution of values. Differences in PC distribution were determined by Chi-squared test, whereas PC lengths and related parameters were analyzed by non-parametric Mann-Withney *U*-test and Spearman’s correlation analysis. *P*-values < 0.05 were considered significant.

## Results

### Reduction of Striatal DAT Expression Without Alteration of D2R and TH Levels in *Npc1^nmf*164*^* Mice

**FIGURE 1:**
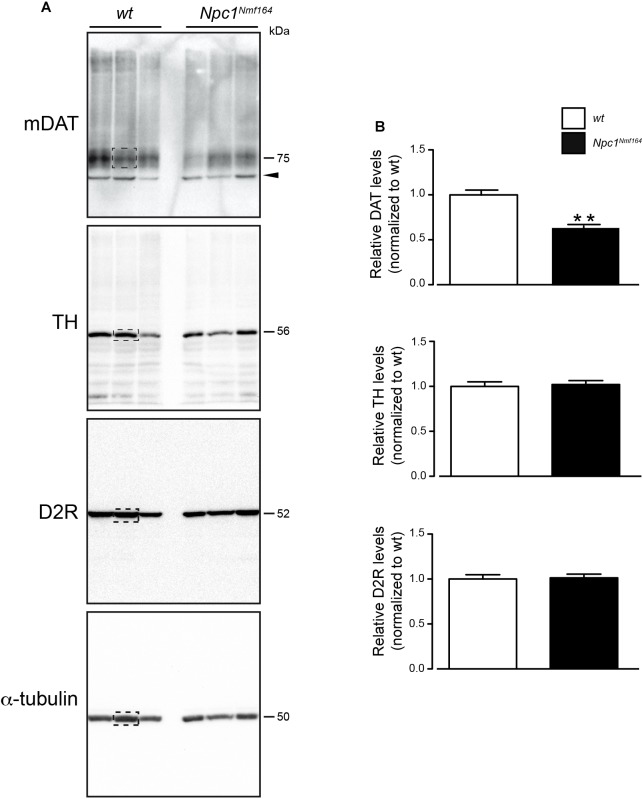
*Npc1^nmf*164*^* mice display reduction of striatal mDAT expression. **(A)** Western blot analysis of DAT, D2R, TH, and α-tubulin in representative dorsal striatum (DS) samples of *wt* or *Npc1^nmf*164*^* juvenile littermate mice. Boxed areas highlight bands of interest. **(B)** Densitometric quantification of immunostained mDAT, D2R, and TH proteins in DS extracts prepared from juvenile *wt* or *Npc1^nmf*164*^* littermate mice are shown as median ± SEM (*n* = 5 mice per group), ^∗∗^*P* < 0.01, Mann-Withney *U*-test. Arrowhead indicates likely immature form of DAT.

It is know the functional relation between DAT and TH expression in the striatum (Salvatore et al., 2016) and the physical association between D2R and DAT proteins at striatal presynaptic terminals (Lee et al., 2007). To examine whether the dysregulation of cholesterol homeostasis in *Npc1*-mutant mice affects dopaminergic signaling in DS, Western blot analysis for the mature form of DAT (mDAT), D2R, TH was performed on protein extracted from tissue punches of PN30 *Npc1^nmf*164*^* mice, and *wt* littermates (Figure 1A). Only striatal mDAT levels were found significantly reduced in *Npc1^nmf*164*^* mice compared to control littermates (*P* < 0.01; Figure 1B).

### 3D Analysis of Striatal PC: Novel Measurement Method Displays Comparable Average Length of PC but a Different Length Frequency Distribution

**FIGURE 2:**
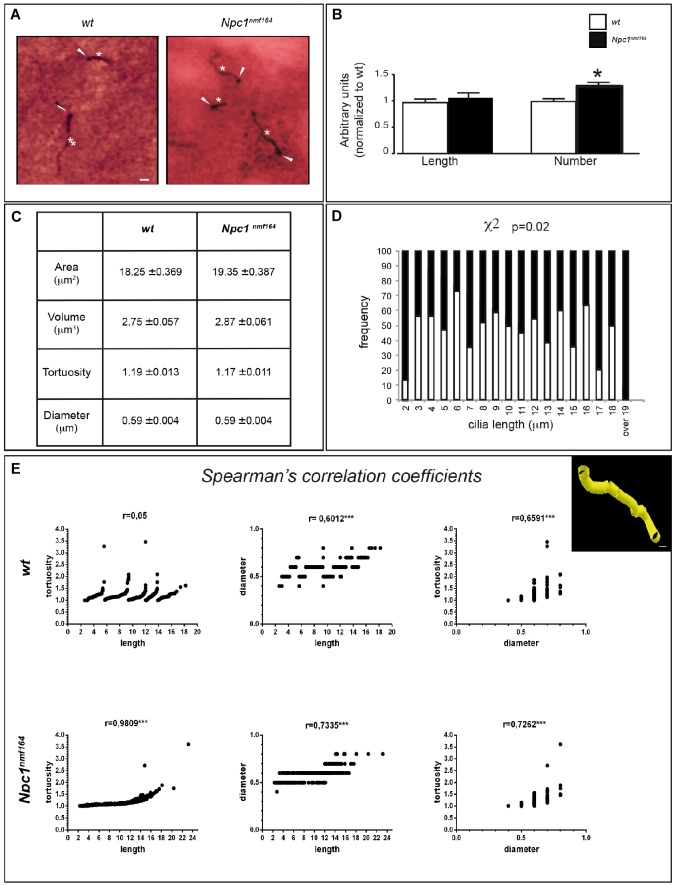
*Npc1^nmf*164*^* mice show an altered number and length distribution of striatal neuronal primary cilia. **(A)** Detection of primary cilia (PC) in the DS of PN30 *wt* and *Npc1^nmf*164*^* mouse by double IHC with antibodies against γ-tubulin (basal body) and adenylyl cyclase III (ACIII, PC shaft) as indicated by arrowheads and asterisks, respectively. Scale bar: 5 μm. **(B)** Histograms represent (median ± SE) the quantification of PC length and number in *wt* (empty bars) and *Npc1^nmf*164*^* (full bars) mice (*n* = 5 animals/genotype). Asterisks indicate statistically significant differences (^∗^*P* < 0.05, Mann-Withney *U*-test and Student’s *t*-tests). **(C)** Summary of ciliary morphological features and corresponding average values, indicated as median ± SE. No significant differences were found. **(D)** Histograms show the significant difference in the distributions of ACIII-positive ciliary length values in the dorsal striatum, between *wt* and *Npc1^nmf*164*^* mice (*P* = 0.02). **(E)** Scatter plot and Spearman’s correlation coefficients between three morphological features (ciliary length, tortuosity and diameter) for each genotype (^∗∗∗^*P* < 0.0005). The upper right panel shows a representative 3D Neurolucida reconstruction of dorsal striatal PC. Scale bar: 1 μm.

*Npc1^nmf*164*^* mice show a reduction of mDAT expression that is likely associated to increased dopaminergic stimulation, which controls PC length through the cAMP pathway (Neve et al., 2004; Ou et al., 2009). To study possible variations of PC morphology in striatal sections from *Npc1^nmf*164*^* and *wt* mice, we performed a double IHC labeling with antibodies against γ-tubulin (as basal body marker) and ACIII (as neuronal ciliary shaft marker) (Figure 2A) coupled to the Neurolucida acquisition system. The latter allows the application to PC analysis of standard tools used for neuron tracing, as simultaneous and precise 3D measurements (±0.5 μm) of length, diameter, and tortuosity.

The experimental results revealed no significant difference between the two genotypes in the mean length of neuronal PC (Figure 2B), as well as in other parameters, including ciliary area, volume, tortuosity, and diameter (Figure 2C). In *Npc1^nmf*164*^* mice, however, significant differences were observed in the number of ACIII-positive PC (*P* = 0.03) and distribution of PC lengths (*P* = 0.02), with an increment of PC with very short (2 μm), or very long length (17 μm and over), compared to control mice (Figure 2D).

Finally, Spearman’s correlation analysis was used to investigate the relationships between three major PC morphological parameters: length, diameter, and tortuosity. The analysis between ACIII-positive PC length and tortuosity, defined as greater bends or kinks in the ciliary axoneme, surprisingly demonstrated a perfect Spearmann’s correlation in *Npc1^nmf*164*^* mice, in contrast to *wt* mice. In mutant mice, in fact, the tortuosity progressively rises with increasing length, whereas in *wt* mice a considerable tortuosity variability is observed both in long and short cilia. Concerning length-diameter and diameter-tortuosity relationships, similar positive Spearman coefficients were found in both *wt* and mutant samples (Figure 2E).

## Discussion

The prominent feature of the NPC1 disease is a distinctive progressive neurodegeneration, with cerebellum and Purkinje neurons being particularly vulnerable (Higashi et al., 1993; Nusca et al., 2014). Some clinical features, however, overlap between lysosomal storage disorders as NPC1 and Parkinson’s disease, suggesting that the two disorders may be pathogenically linked (Storch et al., 2004; Deng et al., 2015).

To characterize the likely contribution of striatal component in the etiology of NPC1 disease, we analyzed the expression of effectors of DA signaling, including mDAT, D2R, and TH by Western Blot analysis of striatal samples from *Npc1^nmf*164*^* mice at a juvenile, asymptomatic age in comparison to *wt* littermates. The reduction of mDAT protein levels in PN30 *Npc1^nmf*164*^* mice is in agreement with the marked symmetrical loss of striatal DAT, especially in the putamen, observed in NPC1 patients by DAT-scan analysis (Terbeek et al., 2017; Tomic, 2018).

The absence of D2R-mediated stimulation increases cAMP level, which in turn leads to neuronal PC elongation (Besschetnova et al., 2010; Miyoshi et al., 2014). It is also known that PC length and density exhibit brain region-specific changes (Sipos et al., 2018) and ciliary D1-receptor translocates to and from cilia in response to environmental cues (Domire et al., 2011). In addition, DAT, TH, and D2R proteins colocalize in nigrostriatal terminals and their expression levels are often affected in neurological/ neurodegenerative disorders. The distribution of D1R and D2R varies along the rostro-caudal axis of the DS (Gangarossa et al., 2013), whereas DAT and D2R directly interact to facilitate the recruitment of DAT to the plasma membrane (Lee et al., 2007).

We have recently reported that there is a reduction of PC density and length in hippocampal neurons of *Npc1*^−/−^ mice as well as in fibroblasts of NPC1 patients, with associated dysregulation of expression/subcellular localization of Shh pathway components (Canterini et al., 2017). As no previous information was available on *Npc1* deficiency-dependent morphological changes of striatal PC, we performed a 3D analysis of ciliary images for understanding structural determinants of normal and pathological PC function.

The remarkable length of PC of striatal neurons of either *wt* or *Npc1^nmf*164*^* mice is in agreement with a previous study that reported the presence in the striatum of a large number of long ACIII-positive PC (Bishop et al., 2007). The absence of statistically significant differences between the two genotypes in the average values of ACIII-, γ tubulin-positive PC length, and related parameters indicates that the mild alteration of DAT expression that we found does not lead to a structural remodeling of dorsal striatal PC in mutant mice. However, a more detailed analysis showed that *Npc1^nmf*164*^* mice display an increased number of ACIII-, γ-tubulin-positive PC and a different distribution of their lengths, together with increased tortuosity in a length-dependent manner, suggesting anomalies of ciliary functions.

The wider range of lengths and the positive correlation between length and tortuosity observed in *Npc1^nmf*164*^* suggest that mutant cilia are “unstable.” Such instability possibly reflects a mis-regulation of axonemal length. Mutant cilia could undergo excessive elongation and fragmentation that would explain the increment of either very short or very long PC, which is observed in mutant mice. Similar ciliary instability was reported in Kdm3a mutants (Yeyati et al., 2017).

The reason of regional difference of PC expression in NPC1 disease is still unclear. It could be attributable to multiple factors such as regional changes in dopaminergic signaling or projections, spatial regulation of Shh released from dendrites and axons of dopaminergic neurons or to differences in intracellular cAMP levels that positively regulate the length of PC through the modulation of protein kinase A activity.

In conclusion, our findings identify for the first time subtle changes occurring in the striatum of juvenile asymptomatic *Npc1^nmf*164*^* mice that could contribute to NPC1 disease neurological manifestations. This is in agreement with: (i) our previous studies that demonstrated early developmental defects which occur postnatally in the cerebellum of *Npc1*-deficient mice and largely anticipate motor deficits, typically observed during adulthood (Nusca et al., 2014; Caporali et al., 2016); (ii) reported embryonic abnormalities in the metabolism of cholesterol in striatal neurons of *Npc1-*deficient mice (Henderson et al., 2000); (iii) DAT KO-mice display ataxic symptoms, tremors, dystonia and saccade-failure (Cyr et al., 2003), typical of related-dopamine-transporter-deficiency syndrome (Ng et al., 2014) and late-onset NPC1 disease (Vanier, 2010).

Although the anomalies in DAT expression and PC of Pn30 Npc1 mice we report in this study appear mild, we expect later stages of the disease to be landmarked by more robust alterations, with a decrement of DAT expression and PC length, as consequence of a progressive worsening of the perturbations of plasma membrane lipid content (Peake and Vance, 2010).

## Ethics Statement

Animal experimental protocols and related procedures were approved by the Italian Ministry of Health-General Directorate of Animal Health (995/2016; D.Igs. 26/2014). All efforts were made to minimize animal suffering, according to European Directive 2010/63/EU.

## Author Contributions

ML performed IHC, image acquisition, and statistical data-analysis. CDP and GLS performed Western Blot and statistical data-analysis. MF contributed with advise and discussion. SC conceived the project. SC and DM directed the project, prepared the figures, and wrote the manuscript.

## Conflict of Interest Statement

The authors declare that the research was conducted in the absence of any commercial or financial relationships that could be construed as a potential conflict of interest.
