# Supplementary material for: Anomalies in Dopamine Transporter Expression and Primary Cilium Distribution in the Dorsal Striatum of a Mouse Model of Niemann-Pick C1 Disease
Source: Front Cell Neurosci. 2019 May 24;13:226. doi: 10.3389/fncel.2019.00226 (PMC6544041; doi:10.3389/fncel.2019.00226)
Supplement: Supplementary file 1 [file Table_1.DOCX]

Supplementary Material

## Supplementary Table 1

The bodies and brains of Npc1^nmf164^ wt and mutant mice of both sexes were weighed at PN30.

| Age (days) | Genotype | Body weight (g) | Brain weight (g) |
| --- | --- | --- | --- |
| 30 | *Npc1^nmf164^*(+/+) | 14.10 (n=14) | 0.408 |
| 30 | *Npc1^nmf164^* (-/-) | 12.81 (n=10) | 0.374 |

**
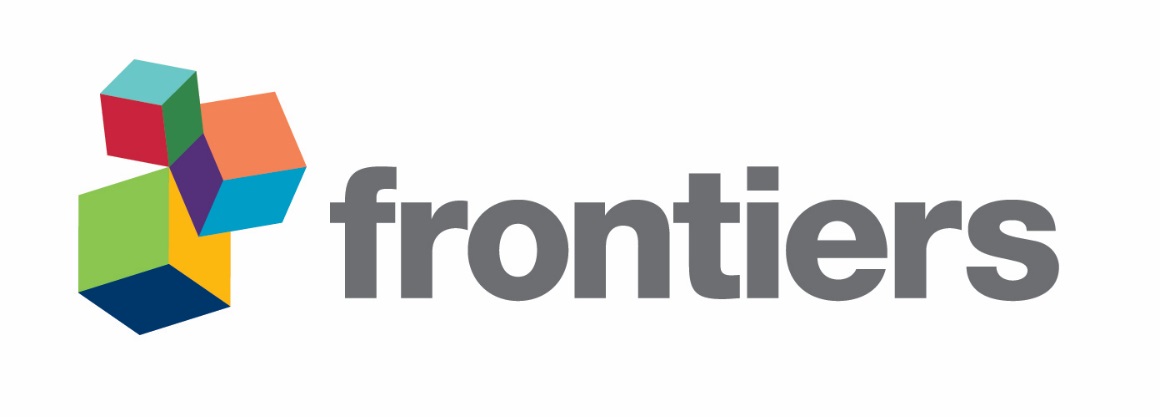
**
