# Supplementary material for: Anomalies in Dopamine Transporter Expression and Primary Cilium Distribution in the Dorsal Striatum of a Mouse Model of Niemann-Pick C1 Disease
Source: Front Cell Neurosci. 2019 May 24;13:226. doi: 10.3389/fncel.2019.00226 (PMC6544041; doi:10.3389/fncel.2019.00226)
Supplement: Supplementary file 2 [file Table_2.DOCX]

Supplementary Material

## Supplementary Table 2

List of antibodies used in western blot analysis and immunohistochemistry.

| **Primary antibodies/Origin** | **Dilution** | **Producer, Catalog #** |
| --- | --- | --- |
| Anti-dopamine transporter (DAT)/ rat | 1:1000 | Millipore, #MAB369, |
| Anti-dopamine receptor 2 (D2R)/ rabbit | 1:1000 | Millipore, #AB5084P |
| Anti-tyrosine hydroxylase (TH)/ mouse clone LNC1 | 1:2000 | Chemicon, #MAB318 |
| Anti-α-tubulin/ mouse clone DM1A | 1:1000 | Sigma-Aldrich, #T9026 |
| Anti-adenylyl cyclase III (ACIII)/ rabbit | 1:150 | Santa Cruz Biotech, #SC-588 |
| Anti-γ-tubulin/mouse | 1:4000 | Sigma-Aldrich, #T6557 |
| **Secondary antibodies** |  |  |
| Horseradish peroxidase-conjugated anti-mouse IgG | 1:2000 | Amersham Biosciences, #NA931 |
| Horseradish peroxidase-conjugated anti-rabbit IgG | 1:1000 | Cell Signaling Technology, #7074 |
| Horseradish peroxidase-conjugated anti-rat IgG | 1:1000 | Amersham Biosciences, #NA935 |
| Biotinylated anti-rabbit IgG | 1:200 | Vector Laboratories, #PK-6101 |
| Biotinylated anti-mouse IgG | 1:200 | Vector Laboratories, #PK-6102 |

**
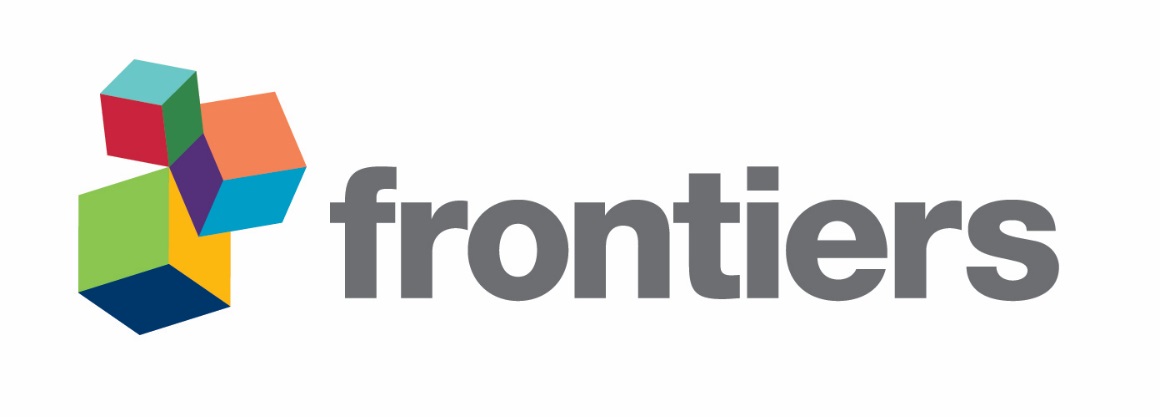
**
